# Nutritional status of children 0–59 months in selected intervention communities in northern Ghana from the africa RISING project in 2012

**DOI:** 10.1186/s13690-016-0124-1

**Published:** 2016-04-04

**Authors:** Mary Glover-Amengor, Isaac Agbemafle, Lynda Larmkie Hagan, Frank Peget Mboom, Gladys Gamor, Asamoah Larbi, Irmgard Hoeschle-Zeledon

**Affiliations:** Council for Scientific and Industrial Research-Food Research Institute, Box M 20, Accra, Ghana; School of Public Health, University of Health and Allied Sciences, PMB 31, Ho, Ghana; Department of Family and Consumer Sciences, Faculty of Agriculture, University for Development Studies, Nyankpala, Tamale, Ghana; International Institute of Tropical Agriculture, Africa Research in Sustainable Intensification Project, Box TL 6, Tamale, Ghana; International Institute of Tropical Agriculture, Africa Research in Sustainable Intensification Project, PMB 5320, Oyo-Road, Ibadan, Nigeria

**Keywords:** Wasting, Stunting, Underweight, Children, Northern Ghana, Africa RISING

## Abstract

**Background:**

Poor nutritional status during childhood and its long-term impact on economic growth and wellbeing is well known. This study assessed the nutritional status of children in selected communities in northern Ghana, to serve as baseline data for the Africa Research in Sustainable Intensification for the Next Generation (Africa RISING) project that sought to improve farm-household nutrition through agriculture.

**Methods:**

A cross-sectional study was conducted among children 0–59 months in selected communities in the Northern (*Tibali* and *Cheyohi No. 2*), Upper West (*Goli* and *Zanko*) and Upper East (*Bonia* and *Sambulgu*) regions of northern Ghana. A pre-tested, semi-structured questionnaire was used to obtain information on background characteristics of caregivers and children. Weight and height were measured for children following World Health Organization (WHO) procedures and transformed into z-scores using the WHO Anthro.

**Results:**

All the caregivers (522) were females; majority (73.4 %) had no formal education, 82.7 % were married and 70.5 % engaged in farming. In all, 533 children were recruited: Northern region (38.6 %), Upper West (33.4 %) and Upper East (28.0 %). Majority (52.5 %) of the children were males. The mean age was 32 ± 19 months. Levels of stunting, underweight and wasting were 27.2, 17.6 and 8.2 % respectively. Stunting, underweight and wasting levels increased within the first two years of life. Overall, 33.8 % of the children in northern Ghana were malnourished; 20.2 % were from the Northern region, 7.0 and 6.8 % were from Upper East and Upper West respectively.

**Conclusion:**

Different forms of malnutrition still exist as a public health problem in various communities in northern Ghana and need to be curtailed using effective agriculture-nutrition sensitive interventions.

## Background

Childhood undernutrition in the form of chronic and acute malnutrition coexist in many populations in developing countries. Unlike acute malnutrition which is associated with immediate crisis such as periodic food shortages, chronic malnutrition is due to inadequate nutrition over a prolonged period resulting from latent poverty, chronic food insecurity, poor feeding practices and repeated episodes of health problems (such as infections) or poor health services in an unhealthy environment [1, 2]. Recent estimates indicate that, 165 million of all children under 5 years worldwide are stunted and a further 52 million are wasted; Africa and Asia have the highest burden [3]. Underweight, wasting and stunting respectively contribute to 19.0, 14.6 and 14.5 % of global deaths [4]. Vitamin A and zinc deficiencies contribute substantially to micronutrient deficiency related-deaths whilst iodine and iron deficiencies coupled with stunting alone, contribute to children not reaching their full developmental potential [3]. Also children who survive malnourished childhood are less productive physically and intellectually, and are more prone to chronic illness and disability in adult life [5, 6].

In Ghana, reports on child undernutrition are not different. Statistics indicates that 28 % of children under 5 years were stunted and 9 % were wasted [7]. A 2012 report by the Ghana statistical service indicated that the stunting prevalence in northern Ghana ranged from 31.5 to 37.4 % [8]. This can be mainly attributed to food insecurity issues as well as poor child and maternal care in addition to poor nutrition. In the three northern regions of Ghana, undernutrition is high relative to the south and about 16 % (680,000 people) of households are severely or moderately food insecure [9]. These alarming undernutrition and hunger estimates in northern Ghana have directed focus of some Non-governmental organizations (NGOs) in northern Ghana to agriculture-nutrition sensitive programmes. The Africa Research in Sustainable Intensification for the Next Generation (Africa RISING) is one of such agriculture-nutrition sensitive programmes in northern Ghana: as part of the U.S. government’s “Feed the Future” initiative. Through action research and development partnerships (International Institute of Tropical Agriculture, West Africa and International Livestock Research Institute, East Africa), Africa RISING will create opportunities for smallholder farm households to move out of hunger and poverty through sustainably intensified farming systems that improve food, nutrition, and income security, particularly for women and children. This study was aimed at benchmarking the nutritional status of children in selected communities in northern Ghana, to serve as baseline data for the Africa RISING project to be implemented in those communities. Additionally, the predictors of nutritional status of children in these communities were also determined.

## Methods

### Design and setting

This was a cross-sectional study conducted among children 0–59 months in northern Ghana (Northern region, Upper East region and Upper West region). Two districts in each region, were selected from intervention sites mapped out by the Africa RISING project. The districts were Wa West and Nadowli districts (Upper West Region), Kassena Nankana and Bongo districts (Upper East Region) and Kumbugu and Savelugu districts (Northern Region). In each selected district, a rural and peri-urban community were selected. The communities selected in Wa West and Nadowli districts were *Zanko* and *Goli* respecively, in Kassena-Nankana and Bongo districts they were *Bonia* and *Sambulgu* respecively, while *Tibali* and *Cheyohi No. 2* were the communities selected for Kumbugu and Savelugu districts respectively.

### Sampling

A simple population proportion formula assuming a stunting prevalence of 35 % in northern Ghana, alpha level of 0.05 and 80 % power was used to obtain a sample size of 513 and this was rounded up to 522 to improve the precision of the estimates [8, 10]. Two communities were purposively selected from each district using the criteria of market access and duration of planting season. All communities in the selected districts were listed and classified as either small (≤45 hamlets), medium (46–100 hamlets) and large (>100 hamlets). In the small communities, all households were sampled, but with the medium and large communities, probability proportionate to size was used to select the required number of households. Thus in *Cheyohi No. 2* and *Zanko*, all households were sampled. In the remaining communities, the number of households sampled was randomly selected based on their respective populations. Caregivers and their children were recruited from each household. A caregiver was defined as a person responsible for the preparation of the family meal during the interview week. In compounds with multiple households, households were selected by balloting and all children 0–59 months in the selected household were included in the study. Caregiver and child consent was obtained from each participant recruited into the study. The study protocol was approved by the Institutional Review Board (IRB) of the Council for Scientific and Industrial Research (CSIR), Accra, and permission also was sought from community leaders and chiefs as well as household heads.

### Data collection

Information on participants’ background characteristics was obtained using a pretested questionnaire. Questions on sex, age, caregiver’s level of education, marital status, household size and occupation were obtained from the respondents through one-on-one interview. Nutritional status of children aged 0–59 months in the selected households were determined by taking anthropometric measurements based on WHO standard procedures [11]. Body weight was measured using an electronic digital scale (Tanita Electronic scale BWB-800). Length and height measurements were taken using an infantometer (Seca 416) and stadiometer (Seca 207) respectively. Oedema was assessed in all children recruited. All children’s birth records were assessed from their birth certificates and weighing cards. The interviewers were trained on collecting anthropometric measurements. Questionnaire was pre-tested and equipment were calibrated before use. After data collection, the questionnaire were cross-checked in the field and all necessary corrections made.

## Statistical analysis

Data was analysed using SPSS version 20.0 and WHO Anthro. The WHO Anthro was used to convert weight, height and age of child (months) into weight-for-age z-score (WAZ), weight-for-height z-score (WHZ) and height-for-age z-score (HAZ). Children whose full birth date (month and year) were not obtained, and children whose weight and/or height were not measured were excluded from one or more of the anthropometric indices. There were no flagged records and oedema was not present in any of the children assessed; hence these were not controlled for in the anthropometric indices. Anthropometric classifications were based on global standards: <−3SD, <−2SD and ≥ −2SD [11]. Children whose HAZ, WAZ and WHZ were below minus two standard deviations (−2 SD) from the median of the reference population were considered as stunted, underweight and wasted respectively. The three anthropometric indices were combined to determine the overall nutritional status. A child with all three anthropometric indices < −2SD or ≥ −2SD was classified as severely malnourished or well-nourished respectively. If two of the three anthropometric indices were < −2SD, the child was considered moderately malnourished and when one of the three anthropometric indices was < −2SD, the child was considered marginally malnourished.

Descriptive statistics was performed for the socio-demographic characteristics of the caregivers and children. Chi-square analysis was used to examine associations between background characteristics of the children and their levels of stunting, wasting and underweight. Analysis of variance (ANOVA) was used to test for differences in mean HAZ, WAZ and WHZ by the children’s background characteristics. Binary logistic regression analyses were conducted to estimate adjusted odds ratio and 95 % confidence intervals to determine the predictors of poor nutritional status. The dependent variable was overall nutritional status (0 = well-nourished and 1 = malnourished). The independent variables were caregiver characteristics such as age, sex, educational level, occupation, marital status, region, household size, feeding frequency and primary source of obtaining food. The other independent variables were the nutritional status of caregivers (body mass index) and child characteristics namely sex of child and age of child. A *p*-value < 0.05 was considered significant.

## Results

### Background characteristics

A total of 522 households in northern Ghana participated in this study. Most (68.2 %) of the households were headed by male heads but all the caregivers were females. Majority (69.9 %) of the caregivers had no formal education, 73.6 % were married and 66.1 % were farmers (Table 1). Apart from farming, caregivers engage in other economic activities such as trading, fishing, shea butter production, fermented maize brewing and skilled artisanship? (33.9 %). Most households (47.5 %) were 6–10 in number and the average household size was 7. Monthly income was below 100 cedis for 64.9 % of the caregivers, 27.6 % of households ate twice a day and 58.4 % of foods eaten were produced by caregivers themselves (Table 1).

**Table 1:** Background characteristics of caregivers in northern Ghana from the Africa RISING project in 2012 (*N* = 522)

Characteristics	Regions			
	Northern (*n* = 113) *n* (%)	Upper West (*n* = 271) *n* (%)	Upper East (*n* = 138) *n* (%)	Total (*N* = 522) *n* (%)
Sex of household head				
Female	5 (4.4)	112 (41.3)	56 (40.5)	173 (33.1)
Male	108 (95.6)	159 (58.7)	82 (59.5)	349 (68.2)
Age (years)				
15–19	2 (1.8)	18 (5.9)	3 (1.5)	23 (3.9)
20–29	35 (31.0)	71 (26.4)	28 (20.4)	134 (25.8)
30–39	38 (33.6)	73 (27.1)	51 (37.2)	162 (31.2)
40–49	25 (22.1)	51 (19.0)	29 (21.2)	105 (20.2)
≥ 50	13 (11.5)	58 (11.2)	27 (19.7)	98 (18.9)
Educational level				
None	106 (93.8)	147 (54.2)	112 (81.2)	365 (69.9)
Primary	4 (3.5)	54 (19.9)	11 (8.0)	69 (13.2)
Junior high/middle school	1 (0.9)	49 (18.1)	15 (10.9)	65 (12.5)
Senior high school +	2 (1.8)	21 (7.7)	0 (0.0)	18 (4.5)
Marital status				
Single	12 (10.6)	26 (9.6)	14 (10.1)	52 (10.0)
Married	95 (84.1)	177 (65.3)	112 (81.2)	384 (73.6)
Divorced	1 (0.9)	6 (2.2)	0 (0.0)	7 (1.3)
Separated	0 (0.0)	5 (1.8)	2 (1.4)	7 (1.3)
Widowed	5 (4.4)	57 (21.0)	10 (7.2)	72 (13.8)
Household size				
≤ 5	17 (15.0)	119 (43.9)	45 (32.6)	181 (34.7)
6–10	39 (34.5)	130 (48.0)	79 (57.2)	248 (47.5)
≥ 10	57 (50.4)	22 (8.1)	14 (10.1)	93 (17.8)
Occupation				
Farming	63 (55.8)	184 (67.9)	98 (77.8)	345 (66.1)
*Other activities	50 (44.2)	87 (32.1)	40 (29.0)	177 (33.9)
Income (GH¢)				
<100	87 (77.0)	135 (49.8)	117 (84.8)	339 (64.9)
101–300	23 (20.4)	91 (33.6)	19 (13.8)	133 (25.5)
301–500	2 (1.8)	20 (7.4)	0 (0.0)	22 (4.2)
>500	1 (0.9)	25 (9.2)	2 (1.4)	28 (5.2)
Meal frequency in a day	109	267	130	506
Once	4 (3.5)	7 (2.6)	12 (8.7)	23 (4.4)
Twice	2 (1.8)	75 (27.7)	67 (48.6)	144 (27.6)
Thrice	101 (89.4)	184 (67.9)	58 (42.0)	343 (65.7)
Four times	6 (5.3)	5 (1.8)	1 (0.7)	12 (2.3)
Primary source of food				
Own production	81 (71.7)	144 (53.1)	80 (58.0)	305 (58.4)
Purchases	3 (2.7)	22 (8.1)	9 (6.5)	34 (6.5)
Borrowed/battered	1 (0.9)	0 (0.0)	1 (0.7)	2 (0.4)
Food aid	0 (0.0)	1 (0.4)	1 (0.7)	2 (0.4)
Own production + purchases	28 (24.8)	104 (38.4)	47 (34.1)	179 (34.3)

*Other activities = > Other activities include trading, fishing, share butter production, fermented millet “pito” brewing and skilled artisan

In all, 533 children were recruited: *Bonia* (23.8 %), *Cheyohi No. 2* (14.4 %), *Goli* (14.0 %), *Sambulgu* (16.9 %), *Tibali* (18.4 %) and *Zanko* (12.5 %). *Cheyohi No. 2* and *Tibali* were communities in Northern region (38 %), *Bonia* and *Sambulgu* were communities in Upper East region (29 %) and *Zanko* and *Goli* were communities in Upper West region (33 %). Majority (52.5 %) of the children were males. Children within the ages of 24–35 months (21.9 %) were twice as many as children within the ages of 6–11 months (10.2 %; Table 2). Twenty-five percent of the children constituted the largest age cohort of 48–59 months whilst the least cohort of 10 % was the 0–5 months aged infants. The mean age was 32 ± 19 months. The mean height and weight for the children in this population was 86.2 ± 19 cm and 11.6 ± 3.7 kg. There were no regional differences in background characteristics either among the caregivers or their children.

**Table 2:** Background characteristics of children 0–59 months in northern Ghana from the Africa RISING project in 2012 (*N* = 533)

Characteristics	Regions			
	Northern (*n* = 206) *n* (%)	Upper West (*n* = 178) *n* (%)	Upper East (*n* = 149) *n* (%)	Total (*N* = 533) *n* (%)
Sex				
Male	107 (51.9)	90 (50.6)	83 (55.7)	280 (52.5)
Female	99 (48.1)	88 (49.4)	66 (44.3)	253 (47.5)
*Age (months)				
0–5	29 (14.4)	8 (4.6)	16 (10.5)	53 (10.0)
6–11	23 (11.4)	15 (8.6)	16 (10.5)	54 (10.2)
12–23	32 (15.8)	19 (10.9)	24 (15.8)	75 (14.2)
24–35	46 (22.8)	33 (18.9)	37 (24.3)	116 (21.9)
36–47	32 (15.8)	37 (21.1)	30 (19.7)	99 (18.7)
48–59	40 (19.8)	63 (36.0)	29 (19.1)	132 (25.0)

*Age (months) = > Children whose full birth date (month and year) were not obtained were excluded

### WHZ, HAZ and WAZ profile

The mean WHZ, HAZ and WAZ in northern Ghana were −0.56 ± 1.08, −1.16 ± 1.55 and −1.08 ± 1.16 respectively as shown in Table 3. About 5.6 % of children under five years in northern Ghana have HAZ < −3SD and 21.6 % had HAZ < −2SD. WAZ was < −3SD for 4.3 % of the children whilst 13.4 % of the children had WAZ < −2SD. In terms of WHZ, 2.6 % of the children had WHZ < −3SD whilst 5.6 % had WHZ < −2SD (Table 3). None of the children had HAZ, WAZ and WHZ ≥2SD.

**Table 3:** Children 0–59 months classified according to their height-for-age, weight-for-age and weight-for-height from the Africa RISING project in northern Ghana in 2012

Characteristics	*Height-for-age (HAZ) [*N* = 496]				*Weight-for-age (WAZ) [*N* = 493]				*Weight-for-height (WHZ) [*N* = 499]			
	<−3SD n (%)	<−2SD n (%)	≥ − 2SD n (%)	Mean Z-score (95 % CI)	<−3SD n (%)	<−2SD n (%)	≥ − 2SD n (%)	Mean Z-score (95 % CI)	<−3SD n (%)	<−2SD n (%)	≥ − 2SD n (%)	Mean Z-score (95 % CI)
Sex												
Male	17 (6.5)	49 (18.8)	194 (74.6)	−1.19 (−0.35, 0.20)	13 (5.0)	31 (12.0)	214 (82.9)	−1.07 (−0.19, 0.22)	7 (2.7)	16 (6.2)	237 (91.2)	−0.52 (−0.11, 0.27)
Female	11 (4.7)	58 (24.6)	167 (70.8)	−1.12 (−0.35, 0.20)	8 (3.4)	35 (14.9)	192 (81.7)	−1.08 (−0.19, 0.22)	6 (2.5)	12 (5.0)	221 (92.5)	−0.60 (−0.11, 0.27)
Age (months)												
0−5	3 (5.7)	4 (7.5)	46 (86.8)	−0.32 (−1.08, 0.45)	4 (7.5)	6 (11.3)	43 (81.1)	−0.87 (−1.36, −0.38)	2 (3.8)	6 (11.5)	44 (84.6)	−0.63 (−11.04, −0.21)
6−11	3 (5.6)	4 (7.4)	47 (87.0)	−0.43 (−0.93, 0.07)	4 (7.4)	7 (13.0)	43 (79.6)	−1.23 (−1.59, −0.87)	5 (9.3)	7 (13.0)	42 (77.8)	−1.25 (−1.56, −0.94)
12−23	2 (2.7)	18 (24.3)	54 (73.0)	−1.33 (−1.59, −1.07)	6 (8.1)	12 (16.2)	56 (75.7)	−1.34 (−1.58, −1.09)	3 (4.1)	6 (8.1)	65 (87.8)	−0.94 (−1.17, −0.70)
24−35	7 (6.1)	32 (28.1)	75 (65.8)	−1.45 (−1.67, −1.23)	2 (1.6)	16 (14.0)	96 (84.2)	−1.06 (−1.25, −0.87)	1 (0.6)	6 (5.3)	107 (93.9)	−0.39 (−0.57, −0.20)
36−47	6 (6.1)	25 (25.6)	67 (63.4)	−1.33 (−1.57, −1.09)	3 (3.1)	11 (11.3)	83 (85.6)	−0.94 (−1.15, −0.72)	2 (2.0)	1 (1.0)	94 (96.9)	−0.26 (−0.46, −0.07)
48−59	7 (6.8)	24 (23.3)	72 (69.9)	−1.36 (−1.58, −1.14)	2 (2.0)	14 (03.9)	85 (84.2)	−1.05 (−1.23, −0.87)	0 (0.0)	2 (2.4)	99 (98.0)	−0.36 (−0.52, −0.20)
Region												
Northern	20 (10.0)	64 (31.8)	117 (58.2)	−1.41 (−1.68, −1.14)	19 (9.5)	34 (17.1)	146 (73.4)	−1.38 (−1.57, −1.20)	12 (5.9)	15 (7.4)	175 (86.6)	−0.78 (−0.95, −0.61)
Upper West	3 (2.0)	20 (13.4)	126 (84.6)	−0.85 (−1.06, −0.64)	1 (0.8)	14 (10.9)	133 (103.9)	−0.72 (−0.89, −0.55)	0 (0.0)	7 (4.7)	143 (95.3)	−0.31 (−0.46, −0.16)
Upper East	5 (3.4)	23 (15.8)	118 (80.8)	−1.13 (−1.31, −0.96)	1 (0.7)	18 (12.3)	127 (87.0)	−1.01 (−1.15, −0.87)	1 (0.7)	6 (4.1)	140 (95.2)	−0.50 (−0.66, −0.35)
Total	28 (5.6)	107 (21.6)	361 (72.8)	−1.16 ± 1.55	21 (4.3)	66 (13.4)	406 (82.4)	−1.08 ± 1.16	13 (2.6)	28 (5.6)	458 (91.8)	−0.56 ± 1.08

*HAZ, WAZ and WHZ = > Standards used (GSS, 2011; GSS, GHS, Macro, ICF, 2008; WHO, 1995)

### Nutritional status of the children

The levels of stunting, underweight and wasting in these children were 27.2, 17.6 and 8.2 % respectively (Table 4). More children were likely to be stunted and underweight as compared to children who were wasted. There was no difference in stunting and underweight by sex, but males were more likely to be wasted as compared to females. Children less than 6 months were less likely to be stunted, underweight and wasted as compared to older children (6–35 months; Table 4).

**Table 4:** Percentage of children 0–59 months classified as stunted, underweight and wasted in northern Ghana from the Africa RISING project in 2012

Characteristics	*Stunting (*N* = 496)		*Underweight (*N* = 493)		*Wasting (*N* = 499)	
	Absent	Present	Absent	Present	Absent	Present
Sex						
Male	194 (74.6)	66 (25.4)	214 (82.9)	44 (17.1)	237 (91.2)	23 (8.8)
Female	167 (70.8)	69 (29.2)	192 (81.7)	43 (18.3)	221 (92.5)	18 (7.5)
Age (months)						
0−5	46 (86.8)	7 (13.2)	43 (81.1)	10 (18.9)	44 (84.6)	8 (15.4)
6−11	47 (87.0)	7 (13.0)	43 (79.6)	11 (20.4)	42 (77.8)	12 (22.2)
12−23	54 (73.0)	20 (27.0)	56 (75.7)	18 (24.3)	65 (87.8)	9 (12.2)
24−35	75 (65.8)	39 (34.2)	96 (84.2)	18 (15.8)	107 (93.9)	7 (6.1)
36−47	67 (68.4)	31 (31.6)	83 (85.6)	14 (14.4)	94 (96.9)	3 (3.1)
48−59	72 (69.9)	31 (30.1)	85 (84.2)	16 (15.4)	99 (98.0)	2 (2.0)
Region						
Northern	117 (58.2)	84 (41.8)	146 (73.4)	53 (26.6)	175 (86.6)	27 (13.4)
Upper West	126 (84.6)	23 (15.4)	133 (89.9)	15 (10.1)	143 (95.3)	7 (4.7)
Upper East	118 (80.8)	28 (19.2)	127 (87.0)	19 (13.0)	140 (95.2)	7 (4.8)
Total	361 (72.8)	135 (27.2)	406 (82.4)	87 (17.6)	458 (91.8)	41 (8.2)

*Stunting, underweight and wasting = > absent refers good nutritional status and present refers to all forms of stunting, underweight and wasting based on standards used (GSS, 2011; GSS, GHS, Macro, ICF, 2008; WHO, 1995)

**Fig. 1:**
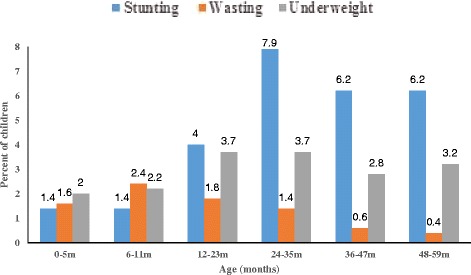
Stunting, underweight and wasting profile across different age groups in northern Ghana from the Africa RISING project in 2012

Stunting and underweight peaked at ages 24–35 months and 12–23 months respectively?. Figure 1 shows that the levels of stunting increased drastically in the second year of life. Levels of underweight increased gradually from birth to when children were 12–23 months old and then levelled off to the third year of life before it started to decline. The levels of wasting peaked when children were about 6–11 months of age and then declineed gradually (Fig. 1). Stunting, underweight and wasting varied by region; it was highest in the Northern region and lowest in the Upper West region (Table 4). The levels of stunting, underweight and wasting in the Northern region was about three times the levels in the Upper West or Upper East region.

Three percent (3.0 %), 12.5 and 18.3 % of the children were severely, moderately and marginally malnourished respectively as shown in Table 5. Overall, 3 out of 10 children (0–59 months) in northern Ghana had some level of malnutrition. Of the 33.8 % of the children with poor nutritional status, 20.2 % were from the Northern region, 7.0 and 6.8 % were from Upper East and Upper West respectively. The overall nutritional status of the females was not different from that of the males. Poor nutritional status increased from birth to the third year of life and then decreased gradually. Poor nutritional status ranged from 3.0 to 8.9 % across the different age groups; the lowest levels were seen in children 0–5 months.

**Table 5:** Nutritional status of children 0–59 months classified according to their anthropometric indices from Africa RISING project in northern Ghana in 2012 (*N* = 499)

Characteristics	Nutritional status				
	Severely malnourished	Moderately malnourished	Marginally malnourished	All forms of malnutrition	Well nourished
Sex					
Male	9 (10.7)	31 (36.9)	44 (52.4)	84 (32.4)	175 (67.6)
Female	6 (7.0)	32 (37.2)	48 (55.8)	86 (35.8)	154 (64.2)
Age (months)					
0–5	2 (13.3)	6 (40.0)	7 (46.7)	15 (28.3)	38 (71.7)
6–11	3 (16.7)	6 (33.3)	9 (50)	18 (32.7)	37 (67.3)
12–23	4 (15.4)	13 (50)	9 (34.6)	26 (34.2)	50 (65.8)
24–35	3 (6.8)	14 (31.8)	27 (61.4)	44 (38.6)	70 (61.4)
36–47	2 (6.1)	11 (33.3)	20 (60.6)	33 (33.7)	65 (66.3)
48–59	1 (2.9)	13 (38.2)	20 (58.8)	34 (33.0)	69 (67.0)
Region					
Northern	14 (13.9)	35 (34.7)	52 (51.5)	101 (49.5)	103 (50.5)
Upper West	0 (0.0)	11 (32.4)	23 (67.6)	34 (22.8)	115 (77.2)
Upper East	1 (2.9)	17 (48.6)	17 (48.6)	35 (24.0)	111 (76.0)
Total	15 (8.8)	63 (37.1)	92 (54.1)	170 (34.1)	329 (65.9)

The chi-square analysis presented in Table 6 show that, household size, region and frequency of feeding were significantly associated with poor nutritional status of children (malnourished children) (*p*-value < 0.05). There were regional differences in the proportion of children who were malnourished. Children from Northern and Upper East region were about seven times (7x) and three times (3x) more likely to be malnourished as compared to children from Upper East region (*p*-value = 0.13; Table 7). Table 7 presents the binary logistic regression results of predictors of the effect of caregiver characteristics on the likelihood of a child being malnourished. Controlling for all possible confounders, caregiver’s occupation was a significant predictor of poor nutritional status. Children’s whose caregivers were farmers were four times more likely to be malnourished as compared to children whose caregivers were traders (OR = 4.15; 95 % CI = 1.17–14.67).

**Table 6:** Associations between child nutritional status and caregiver characteristics from the Africa RISING project in northern Ghana in 2012^#^

Characteristics	Nutritional status		*p*-value
	Malnourished	Well nourished	
Sex of household head			
Female	32 (27.8)	83 (72.2)	0.05
Male	144 (38.2)	233 (61.8)	
Age group of caregivers (years)			
15–19	4 (26.7)	11 (73.3)	0.50
20–29	51 (36.7)	88 (63.3)	
30–39	73 (39.0)	114 (61.0)	
40–49	22 (28.2)	56 (71.8)	
≥50	26 (35.6)	47 (64.4)	
Household size			
≤5	34 (26.8)	93 (73.2)	0.01*
6-10	71 (34.8)	133 (65.2)	
>10	71 (44.1)	90 (55.9)	
Marital status			
Unmarried	18 (27.3)	48 (72.7)	0.13
Married	158 (37.1)	268 (62.9)	
Region			
Northern	101 (51.0)	97 (49.0)	<0.0001*
Upper West	42 (28.8)	104 (71.2)	
Upper East	33 (22.3)	115 (77.7)	
Educational level			
None	136 (38.2)	220 (61.8)	0.07
Primary and above	40 (29.4)	96 (70.6)	
Occupation			
Farming	110 (34.1)	213 (65.9)	0.28
Trading	66 (39.1)	103 (60.9)	
Income (GH¢)			
<100	119 (34.8)	223 (65.2)	0.78
101–300	47 (38.5)	75 (61.5)	
301–500	7 (41.2)	10 (58.8)	
>500	3 (27.3)	8 (72.7)	
Primary source of obtaining food			
Own production	117 (38.4)	188 (61.6)	0.28
Purchases	6 (28.6)	15 (71.4)	
Own production + purchases	53 (31.9)	113 (68.1)	
Feeding frequency			
Once	1 (16.7)	5 (83.3)	0.002*
Twice	26 (22.8)	88 (77.2)	
Thrice	149 (40.1)	223 (59.9)	
Body mass index (BMI)			
Underweight	23 (41.8)	32 (58.2)	0.43
Normal	132 (35.9)	236 (64.1)	
Overweight/obese	21 (30.4)	48 (69.6)	

#Children and mothers with incomplete data were excluded; *Significant at *p*-valve < 0.05

**Table 7:** Predictors of poor nutritional status of children from the Africa RISING project in northern Ghana in 2012

Variables	OR	95 % confidence interval		*p*-value
		Lower	Upper	
Sex of child				0.21
Female	0.54	0.20	1.43	
Male	1.00			
Age of child (months)				0.91
0–5	2.22	0.14	34.82	
6–11	1.40	0.19	10.63	
12–23	1.17	0.20	6.92	
24–35	1.12	0.26	4.83	
36–47	0.58	0.12	2.84	
48–59	1.00			
Sex of household head				0.05
Female	5.34	0.97	29.45	
Male	1.00			
Age group (years)				0.46
15–19	0.13	0.01	2.52	
20–29	1.10	0.20	5.90	
30–39	1.49	0.30	7.33	
40–49	2.46	0.29	20.88	
≥50	1.00			
Household size				0.81
≤5	1.54	0.35	6.93	
6–10	1.41	0.42	4.69	
>10	1.00			
Region				0.13
Northern	6.93	1.05	45.50	
Upper East	2.53	0.58	10.98	
Upper West	1.00			
Marital status				0.29
Unmarried	0.42	0.08	2.09	
Married	1.00			
Educational level				0.77
None	0.84	0.26	2.73	
Primary and above	1.00			
Occupation				0.03*
Farming	4.15	1.17	14.67	
Trading	1.00			
Income (GH¢)				0.74
<100	0.16	0.00	5.82	
101–300	0.14	0.00	5.28	
301–500	0.08	0.00	12.55	
>500	1.00			
Primary source of obtaining food				0.99
Own production	0.84	0.25	2.85	
Purchases	0.06	0.06	7.34	
Own production + purchases	1.00			
Feeding frequency				0.60
Once	0.02	0.00	25.32	
Twice	0.93	0.22	3.84	
Thrice	1.00			
Body mass index (BMI)				0.34
Underweight	4.51	0.52	38.85	
Normal	1.26	0.31	5.14	
Overweight/obese	1.00			

*Significant at *p*-valve < 0.05. Adjusted R^2^ = 0.88; OR’s are adjusted for all variables in the table

## Discussion

### Stunting profiles

Adequate nutrition is important for child growth, health and development. This study used height-for-age, weight-for-height, and weight-for-age indices to assess the nutritional status of children 0–59 months in northern Ghana. The height-for-age index was used as an indicator of linear growth. In northern Ghana, 27.2 % of the children were stunted. This was higher than the national stunting prevalence of 22.7 % [8]. High levels of stunting were found in communities in Northern region whilst the lowest levels were found in Upper West region. This conformed to regional data from northern Ghana which indicated that stunting prevalence was highest in Northern region [8]. An evaluation of baseline “Feed the Future” indicators for northern Ghana also showed a similar trend [12]. This indicates that children in Northern region were more likely to be stunted as compared to children from other regions in northern Ghana. Stunting levels did not differ by sex and this is in agreement with “Feed the Future” baseline evaluation report for northern Ghana [12]. Contrary to our findings, previous nationwide surveys reported that stunting varied with sex [7, 8]. The differences could be attributed to numbers which were relatively small for this study as compared to nationwide surveys.

Stunting in this study was highest in 12–23 months old children as compared to 36–47 months old children reported elsewhere [12]. Stunting was highest at 12–23 months as it is related to the ages at which many children cease to be breastfed and are exposed to contamination from water, food and the environment. These findings are consistent with a universal pattern of steep increase in children’s HAZ from birth to age 23 months [13]. Levels of stunting remained equally high in older children (≥24 months). This is in agreement with a study that showed a continued increase in stunting between ages 24 and 60 months using height-for-age difference (HAD) [14]. The high levels of stunting after 24 months may be indicative of long-term consequences of nutritional insults experienced during the first 1000 days as postulated [14].

Also children who are too short for their age may have been receiving inadequate food over a prolonged period of time. Being stunted could also be attributable to recurrent and chronic illness. High stunting prevalence in younger children 0–23 months and in older cohorts (24–59 months) raises important questions about an additional critical window for nutrition interventions against stunting. An analysis of African data sets showed modest gains between 24 and 48 months even in the absence of interventions [13]. A fairly recent study reported substantial height catch-up between 24 months and mid-childhood, also in the absence of intervention [15]. Probably, the observed catch-up growth was due to benefits of previous nutritional interventions in the first 1000 days [15]. What is not known is the potential benefits of nutritional interventions after 24 months of life as long-term consequences of nutritional insults may or may not be reversible after this period? Adequate care must be taken to argue that 24–59 months presents another window of opportunity for catch-up growth as later nutritional interventions may lead to substantial fat-mass accumulation with increased risk for adult onset chronic diseases [16].

### Wasting profiles

Weight-for-height index measures body mass in relation to body height or length and it is used to describe wasting. Ghana’s wasting prevalence stands at 6.2 % as compared to the level of 8.2 % in this study. Statistics indicated that wasting prevalence was highest in Upper West region [8]. This is at variance to results presented here. Baseline “Feed the Future” report is in consonance with this study findings [12]. Wasting in children is reflective of failure to receive adequate nutrition during immediate crisis such as food shortage. Regions in northern Ghana lie in the Savanna vegetation zone with prolonged dry conditions throughout the year. Agriculture production is heavily rainfall dependent and the dry seasons are characterized by non-farming activities such as hunting, local manufacturing, charcoal production, petty trading and wage labour [17]. A report by CARE international indicated that poverty and increasing episodes of hunger occur for 3–5 months annually in northern Ghana [18]. Access to food for these children in poor rural communities may become more challenging with the ever alarming rate of climate change. Thus the vulnerability of children in these communities to food and adequate care may increase. During periods of food insecurity, access to care and dietary intake are compromised. Inadequate care in an unhealthy environment predisposes the child to infections. Infections have been shown to be one of the attributable causes of wasting in children [19, 20]. A study in rural Bangladesh showed high concentrations of acute–phase protein (α-1-acid glycoprotein) and higher gut mucosal damage to be associated with wasting [19]. Infections impair the digestion and absorption of food and can cause further deterioration in wasted children.

Boys are more likely to be wasted than females. This agreed with the results of a national survey in Ghana in 2011 which reported that boys are more likely than girls to be too thin for their height (wasted) [8]. However, an earlier national survey in 2008 did not find any association between wasting and sex of children [7]. Children 0–11 months are more likely to be wasted in comparison to children who are older (≥24 months). This finding agrees with reports of increasing levels of wasting in 0–11 months old children [8, 12]. This confirms the first 1000 days as a critical window of opportunity for nutrition interventions for preventing wasting. A previous longitudinal study in Guatemala demonstrated that growth impact of a high-protein/energy food supplement was largest among children who received the intervention during their first 24–36 months of life compared with children exposed at older months [21]. Nutrition interventions from conception to 24 months have also been shown to have a wide range of physical, cognitive, educational, health and economic productivity outcomes throughout adulthood [22, 23].

The highest level of wasting was seen at 6–11 months. This coincides with the time infants begin to receive foods to complement their breastmilk intake. The age at which breastfed infants are first given complementary foods have been linked to increased risk of diarrhoeal diseases from contaminated weaning foods and a high risk of growth faltering [24]. Additionally, feeding practices such as feeding styles to ensure intake and frequency of feeding are important for preventing wasting. In rural Haryama in India, it was shown that an educational intervention on appropriate complementary feeding practices resulted in a significant but small gains in physical growth [25].

### Underweight and malnutrition profiles

Underweight is reflective of both chronic and acute malnutrition. Levels of underweight (17.6) in these communities studied are higher than the national prevalence of 13.4 % [8]. Levels of underweight were highest in the communities in Northern region whilst the communities in Upper West region had the lowest. Our results were consistent with the most recent national survey [8]. No significant differences were observed for sex as reported elsewhere [12]. The levels of underweight were highest in 12–23 months and 24–35 months old children [8, 12].

Children in selected communities in Northern region were more likely to be malnourished as compared to children from Upper West and Upper East regions. Regional estimates in Ghana indicated that children in Northern region were more likely to be underweight and stunted as compared to children from other regions [7, 8]. This trend was observed even in these selected communities due to the high level of poverty in Northern Ghana. A previous nationwide study in Ghana indicated that children in the poorest households were at least twice more likely to be malnourished in comparison with children from the wealthiest households [8].

### Predictors of poor nutritional status

Sex of child was independently correlated with stunting, wasting and underweight. Few studies in sub-Saharan Africa that have demonstrated sex differences in nutritional status have argued that such differences occur in low socio-economic settings such as northern Ghana [26–28]. Caregivers’ educational level was not a significant predictor of poor child nutritional status. This finding is in agreement with a study conducted among children in Kampala which found that formal education exerted no influence on the nutritional status of children [29]. However, other studies found that a poor educational background could be associated with child undernutrition [30–32]. The differences could be due to the low educational level of the caregivers in this study. Studies have shown that as more caregivers obtain education, the more they will visit a health facility and get some nutrition and health advice from health professionals [33–35]. However, this may not be the case in low-income settings where majority of the caregivers had basic education as their highest level of education.

A World Bank report in 2014 indicated that, teenage caregivers and caregivers in their late 30s, were significantly more likely to have children suffering from all forms of malnutrition. Although not statistically significant, an interesting trend was observed for maternal age. Increasing maternal age was poorly associated with child nutritional status. This is at variance to previously published studies due to the unequal distribution of mothers across the different age groups. Additionally, it could be that older caregivers had less health seeking behaviors as compared to younger caregivers especially in low-income setting like northern Ghana.

Household size was independently associated with poor nutritional status. A study in Ethiopia found that children from larger households were more vulnerable to malnutrition [36]. This could be because food for each household was limited and children were easily affected [37]. However, household size exerted no influence on nutritional status in the binary logistics regression model. Although, other studies [32, 38] have confirmed that household size has no influence on the nutritional status of children, this finding reported here could be due to the large average household size of 5–10 persons in northern Ghana.

Additionally, after adjusting for all possible confounders, caregivers’ occupation was a significant predictor of poor nutritional status. Children whose caregivers were farmers were more likely to be malnourished as compared to children whose caregivers were traders. A study in Ethiopia noted that having a salary from employment (either from wage or government work) was a strong predictor for WHZ and HAZ [33]. This study indicates that having daily income from trading activities was more beneficial as compared to farming in which access to income is seasonal. Also, the poor nutritional status of children whose caregivers are farmers could be due to the fact that the caregivers sell most of their farm produce for money and not for household consumption. Although the caregivers were all smallholder farmers, most of their produce were being sold for money. It was expected that the money obtained from the sale of farm produce may be used to improve the nutritional status of children in the household, but most of the money they earned were used in paying for credits these poor farmers had enjoyed during the lean season (personal observation). Again these caregivers may not have control over their own money made from selling of farm produce so that they can buy a variety of foods, thereby improving dietary diversity of the children within the household. The dynamics of smallholder farmers in northern Ghana may have accounted for the different findings reported in this study.

### Strengths and limitations

This paper established baseline estimates of nutritional status of children 0–59 months in selected communities in northern Ghana from the Africa RISING project in 2012. The cross sectional nature of this study makes causal relationships between maternal and child nutritional status less probable. However, since it was conducted in low-income rural settings like northern Ghana, it will give us an initial point to assess the impact of interventions that would be carried out by Africa RISING in these communities. Further studies on food and nutrition insecurity may be needed in order to identify other determinants of poor nutritional status among children in northern Ghana.

## Conclusion

Different forms of malnutrition still exist as a public health problem in various communities in northern Ghana from the Africa RISING project in 2012. These findings will inform the strategies or priorities of the intervention programme to be implemented by Africa RISING. Also, it will serve as basis to evaluate Africa RISING interventions in these communities. This will inform policies on agriculture-nutrition sensitive interventions to curtail childhood malnutrition.

## Recommendation

Further research on smallholder farmer household food and nutrition security especially of female caregivers is needed to fully understand the dynamics of childhood malnutrition in northern Ghana.

## Abbreviations

WAZ: Weight-for-age z-score
WHZ: Weight-for-height z-score
HAZ: Height-for-age z-score
WHO: World Health Organization
GSS: Ghana Statistical Service
GDHS: Ghana Demographic and Health Survey
MICS: Multiple Indicator Cluster Survey
NGOs: Non-governmental organizations
Africa RISING: Africa Research in Sustainable Intensification for the next generation
IITA: International Institute of Tropical Agriculture
